# P39 ‘Watch’ Out! An audit of piperacillin/tazobactam prescribing in a district general hospital

**DOI:** 10.1093/jacamr/dlag102.045

**Published:** 2026-06-26

**Authors:** Alanna Wyncoll, Vinija Thirucumaran, Abdul Mohamed

**Affiliations:** Bedfordshire Hospitals NHS Foundation Trust, Bedford, UK; Bedfordshire Hospitals NHS Foundation Trust, Bedford, UK; Bedfordshire Hospitals NHS Foundation Trust, Bedford, UK

## Abstract

**Background:**

UK-AWaRe classifies antibiotics into Access, Watch and Reserve use.^1^ Inappropriate use of broad-spectrum antibiotics exacerbates antimicrobial resistance, contributing to unnecessary adverse effects and prolonged admissions. Hospital guidelines individualize drug recommendations to local patterns of resistance,^2^ promoting stewardship and optimizing patient outcomes. Piperacillin/tazobactam, a commonly used broad-spectrum IV antibiotic, falls under the Watch category due to greater resistance potential, requiring careful use for a ‘limited number of infective syndromes’. Restricting its use has been shown to reduce bacterial resistance rates.^3^ In addition, early switch to oral antibiotics limits patient exposure to ‘Watch’ antibiotics, as well as reducing hospital stays, healthcare-associated infections, and expenditure.^4^ The National Antimicrobial IV to oral switch criteria^5^ encourages review and cessation of IV antibiotics as soon as clinically appropriate.

**Objectives:**

To identify opportunities to improve antimicrobial stewardship and prescribing practice by assessing documented indications for initiating piperacillin/tazobactam against local guidelines and timely reviews at 48, 72 and 96 h.

**Methods:**

A snapshot audit was conducted at a District General Hospital, looking at all adult inpatients receiving piperacillin/tazobactam on 11 November 2025 (*n*=41). Patients under intensive care were excluded. Documented indications for initiating Piperacillin/tazobactam were assessed against Trust antimicrobial guidelines. Electronic clinical notes and medication charts were reviewed to identify whether antibiotic reviews occurred at 48, 72, and 96 h post-initiation.

**Results:**

66% of patients were correctly initiated on piperacillin/tazobactam for a first-line indication, with the other 34% of presentations warranting another antibiotic as first-line (predominantly co-amoxiclav in combination with other ‘Access’ antibiotics). The most common incorrect indications were for abdominal sepsis without cholestasis, respiratory sepsis, and community-acquired pneumonia. The majority of inappropriate prescriptions (86%) were started on the day of admission without trial of first-line antibiotics beforehand. At 48 h, 38 patients remained on piperacillin/tazobactam, for which 68% had clearly documented review to continue. At 72 h, 31 patients remained on piperacillin/tazobactam, with 74% reviewed. At 96 h, 27 patients continued, with 70% reviewed. Only 46% of patients had blood cultures taken to guide antibiotic choice. 27% of patients had microbiology input within the 96 h window.

**Conclusions:**

Our results indicate adherence to Piperacillin/tazobactam guidelines could be improved, with better documentation of rationale behind antibiotic choice and continuation. This would aid in preventing overuse of this ‘Watch’ antimicrobial. Considering ‘stop and switch’ could be encouraged at 48 h, with microbiology discussion for patients on prolonged piperacillin/tazobactam courses. These findings and recommendations were disseminated at local Grand Round and departmental teaching. The Trust plans to implement an indication-based prescribing system, which will guide prescribers to first-line choices. The impact of this will be evaluated in a second cycle audit.
